# An investigation of patterns of children's sedentary and vigorous physical activity throughout the week

**DOI:** 10.1186/1479-5868-7-88

**Published:** 2010-12-09

**Authors:** Rebekah M Steele, Esther MF van Sluijs, Stephen J Sharp, Jill R Landsbaugh, Ulf Ekelund, Simon J Griffin

**Affiliations:** 1 Medical Research Council Epidemiology Unit, Cambridge, UK

## Abstract

**Background:**

Participation in higher intensity activity (i.e. vigorous physical activity [VPA]) appears more consistently associated with lower adiposity, unfortunately little is known about the nature and patterns of VPA participation in children.

**Objective:**

To examine the volume and patterns of vigorous and sedentary activity during different segments of the week (weekend, school-based and out-of-school). We also investigated differences by sex, socioeconomic status (SES) and weight status.

**Design:**

A cross-sectional study including 1568 UK children aged 9-10 years.

**Outcome measures:**

Sedentary activity (mins), total activity (counts/min) and VPA (mins) were measured by accelerometry. Using a series of 2 level mixed effects linear regression models we tested differences across the segmented week (school time [0900-1500] vs. out-of-school time [0700-0900 & 1500-2100]; and weekday vs. weekend); all models were adjusted for sex, weight status (gender- and age-specific body mass index [BMI] cut points), SES, age and accelerometer registered wear time.

**Results:**

Boys and girls accumulated higher VPA out-of-school compared to during school (boys mean ± SD 16.9 ± 9.6 vs 12.6 ± 5.8; girls, 13.1 ± 7.7 vs 8.2 ± 4.0, both p < 0.001); but there were no differences for weekday v weekend VPA (p > 0.05). Less time was spent sedentary on weekdays compared to weekends (p < 0.001). Although boys were more physically active and girls accumulated more sedentary time, the overall pattern in which their physical activity intensity varied across the various day segments was similar when stratified by weight status and SES; and large volumes of sedentary time were observed each hour across the day.

**Conclusions:**

The promotion of VPA during the weekend may hold the greatest promise for increasing VPA. Interventions aimed at increasing physical activity in 9-10 year old children should aim to target all children independent of sex, SES or weight status.

## Background

The prevalence of childhood obesity has increased in the UK [1], as it has in the United States [2] and across Europe [3]. Childhood obesity is associated with early markers of adult disease including raised triglycerides, hypertension, impaired glucose tolerance, raised cholesterol and hepatic steatosis [4]. Obesity is also known to track into adulthood [5] and may predispose young people to further diseases later in life [6].

The specific causes of overweight and obesity are varied and complex but, at a population level, are consistent with sustained positive energy balance. Sedentary behaviour and low levels of physical activity may, in part, explain the rising prevalence of childhood overweight and obesity [7]. Current guidelines for health-related physical activity in children recommend at least 60-mins per day of moderate to vigorous intensity physical activity (MVPA) [8]. However, it appears that the magnitude of association between some health outcomes and physical activity is intensity driven, suggesting that higher levels of more intense physical activity may be required for the prevention of obesity and related metabolic risk factors. For example, several studies have reported no association between total and/or moderate physical activity (MPA) and obesity indicators [9,10], whereas participation in higher intensity activity (i.e. vigorous physical activity [VPA]) appears more consistently associated with lower adiposity [9,11,12] and this association may even be independent of time spent sedentary [13]. Unfortunately, little is known about the nature and patterns of VPA participation in children, whereas this information may be able to help identify intervention targets and opportunities. Previous studies investigating physical activity patterns focused on total (expressed as counts per min), combined MVPA, or used self-report sports participation in characterising daily physical activity [14-16]. In addition, they focused on average weekday and weekend activity, [16,17] not hourly patterns, and few have examined these patterns by different groups, defined by, for example, socioeconomic status (SES), sex and weight status (gender- and age-specific body mass index [BMI] cut points).

Reducing sedentary activity has been proposed as a suitable behavioural target for health promotion intervention in children [18]. Whether or not sedentary behaviour displaces physical activity, or more specifically VPA, is a topic of debate. It has been shown that sedentary children (i.e. > 4 hours television viewing) can also engage in large amounts of physical activity [19]. Nevertheless, evidence of the co-occurrence of low levels of activity and high levels of screen time [20], suggests that increased knowledge of patterns of sedentary behaviour may assist in determining opportunities for reducing sedentary behaviour and increasing VPA.

Accelerometry permits greater accuracy and reliability of the assessment of activity compared to self-report [21], and interpretation of the minute-by-minute data enables investigations of segmented patterns of activity (i.e. weekend, school-based, out-of-school activity) and intensity in large samples of children [22]. Therefore, using data from the Sport, Physical activity and Eating behaviour, Environmental Determinants in Young people (SPEEDY) study [23] (a large population based study of 9 and 10 year old British children who all provided accelerometry data), we aimed to investigate the volume and patterns of vigorous and sedentary activity during different segments of the week (weekend, school-based and out-of-school) We also investigated differences in activity behaviour by sex, SES and weight status.

## Methods

### Participants

2064 Year 5 children were recruited from 92 rural and urban schools in Norfolk, England, as part of the SPEEDY study. Data were collected through primary schools during the 12-week summer term of 2007 (April to July). Study design, sampling procedures, selection criteria, participation rates and the data collection procedures have been reported elsewhere [23]. However briefly, schools in the county of Norfolk were sampled purposively to achieve heterogeneity in location, 58.6% of invited schools agreed to participate. At these 92 schools 3619 children were invited to take part, the response rate between schools ranged from 13% to 100%, the overall rate was 57% (n = 2064). Children were given an information pack containing a leaflet for themselves, a letter for their parents/guardians and a consent form. Only children with a fully completed consent form provided on the day of measurement were included in the study. The University of East Anglia local research ethics committee approved the study.

### Physical activity

Free-living physical activity was assessed with the Actigraph activity monitor (GT1 M, Actigraph LCC, Pensacola, US; http://www.theactigraph.com) over one week. Participants were instructed to wear the monitor on an elastic waist band on the right hip during day time except during bathing and other aquatic activities. Activity data were stored at 5-second intervals and were downloaded to a computer upon receipt. Participants who did not manage to record at least 500 mins of activity per day for at least 3 days (including 1 weekend day) were excluded from further analyses. A bespoke programme (MAHUffe; downloadable from: http://www.mrc-epid.cam.ac.uk) was used for data cleaning, reduction and further analyses. Zero activity periods of 10 min or longer were interpreted as "not worn time" and these periods were removed from the summation of activity. Number of minutes per day of valid accelerometer registered wear time was then established. Physical activity variables included average daily counts per minute (counts^.^min^-1^), which is an indicator of overall physical activity; and time (min^.^d^-1^) spent at sedentary and vigorous intensity categories averaged per day over the measurement period. Intensity thresholds applied were <100 counts^.^min^-1 ^(sedentary), >2000 counts^.^min^-1 ^(MVPA) and >4000 counts^.^min^-1 ^(VPA). Our lower limit for the MVPA threshold, corresponds to a walking pace of about 3-4 km/h in children [24], and has previously been used in this age group to study associations between physical activity intensity and adiposity [25].

### Anthropometry and body composition

Non-invasive anthropometry measures were undertaken by trained staff following standardised procedures. Portable Leicester height measures (Seca, Hamburg, Germany) were used to assess height to the nearest millimetre. A non-segmental bio-impedance scale (Tanita, type TBF-300A, Tanita Tokyo, Japan) was used to measure weight (to the nearest 0.1 kilogram) and impedance. Weight status was determined from BMI (weight/height^2^) using gender- and age-specific cut points [26].

### Socioeconomic status (SES)

Parental education was used as a proxy measure for the child's SES. The parent or main carer self-reported their highest educational qualifications (in categories) based upon the education system used in England, Wales and Northern Ireland. Three categories were generated; (i) General Certificate of Secondary Education (academic qualification achieved by secondary students aged 15-16 years [GCSE]) or lower; (ii) General Certificate of Education or equivalent (academic qualification obtained by students in the last two years of secondary education aged 16-18 years [A-level]; (iii) Higher Education (higher level vocational training, university qualification).

### Data Analysis

All analyses were performed using Stata Version 10 (STATA Corp., College Station, Texas, USA). Descriptive statistics were calculated for all variables by sex. The contribution of VPA towards overall MVPA was also estimated as a percentage for boys and girls across the segmented week. Tests for normal distribution revealed that some of the physical activity variables were skewed. Log transformation of these variables resulted in distributions that were more normal. We also repeated the analysis using untransformed outcome variables; where the results were similar to those from the transformed analysis, we present the results from the untransformed analysis for ease of interpretation.

We investigated differences in activity volume and patterns across the week. We used a series of 2 level mixed effects linear regression models to examine differences in 3 different physical activity outcomes (sedentary time (mins), total physical activity (cpm), vigorous physical activity (cpm)) across the segmented week (comparing school time [0900-1500 h], out-of-school time [0700-0900 h & 1500-2100 h] and weekend with each other; and weekday versus weekend). All models were adjusted for sex, weight status, SES, age and accelerometer registered wear time. Individual observations at level 1 were nested within participants at level 2, and lack of independence between observations on the same participant was accounted for by allowing the intercept to vary randomly between participants. Due to the small number of participants in some schools we were unable to account for within school clustering in the main models. However, we dropped schools with fewer than 15 students (5 schools), and fitted 3 level models, with school at level 3. Similar results were observed, so we report only the simpler 2-level model with the larger sample size. To establish whether activity patterns differed by sex, SES or weight status, interaction effects were examined within these models.

To investigate activity patterns in more detail, hourly participation in sedentary and vigorous physical activity was established for the hours between 0700 h and 2100 h. Periods before and after are not shown as the majority of participants recorded no activity during these times. Patterns were investigated separately for boys and girls, different SES groups and weight status groups.

## Results

### Participants

Of the original SPEEDY sample, 2030 (98%) children returned accelerometers following the measurement period, of which the following children were excluded: 45 had faulty monitors; 300 did not record at least 3 days including 1 weekend day; and 117 had missing data for at least one covariate(s). All 1568 remaining children were included for data analysis. Children excluded from the analyses recorded significantly more minutes in VPA than those included (mean ± SD: 28.4 ± 13.3 vs. 25.3 ± 14.1; P < 0.05), no demographic (e.g. sex, SES, age) or anthropometric (e.g. weight, BMI) differences were observed. Table 1 shows the characteristics of the boys and girls included in the analyses. Overall, boys recorded a mean (standard deviation) of 743.7 (62.0) minutes of measurement on weekdays and 697.3 (85.9) minutes at weekends; girls recorded an average of 735.9 (61.9) minutes on weekdays and 682.3 (85.9) minutes at weekends. School-based, out-of-school and weekend VPA accounted for 36%, 32.5% and 32.7% of total MVPA for boys and 32.9%, 29.9%, 30.3% of total MVPA for girls.

**Table 1:** Characteristics of the study population by sex (mean ± SD; n = 1568)

	Boys n = 701	Girls n = 867
Age (y)	10.2 ± 0.3	10.3 ± 0.3
Ethnicity (% non-white)	3.6	3.8
SES - parental education (%)		
- GCSE or lower	35.8	40.0
- up to A level	42.7	39.8
- higher education	21.5	20.2
Height (cm)	141.0 ± 6.5	141.1 ± 6.7
Weight (kg)	35.7 ± 7.5	37.2 ± 8.8^1^
BMI (kg/m^2^)	17.8 ± 2.8	18.5 ± 3.3^1^
Weight status (%)		
- overweight	15.3	19.4^1^
- obese	3.6	6.0
Total PA (counts/min)	716.4 ± 227.1	639.9 ± 215.7^1^
Sedentary (min/day)	450.4 ±56.5	462.7 ± 52.0^1^
Light (min/day)	186.0 ± 32.5	180.1 ± 31.3^1^
Moderate (min/day)	54.2 ± 14.8	44.5 ± 12.3^1^
Vigorous (min/day)	29.7 ± 14.4	21.7 ± 11.2^1^
MVPA (min/day)	84.0 ± 25.8	66.3 ± 20.9^1^
Registered Time (mins)	720.4 ± 61.0	709.1 ± 58.6^1^
Meeting PA recommendations		
- ≥60 min/day MVPA (%)	81.2	60.1^1^

^1^statistical significance between boys and girls (P < 0.01)

Table 2 describes the differences in volumes of physical activity across the segmented week for all participants, and boys and girls separately. The accumulated mean minutes of total physical activity (counts per min), and minutes of VPA out-of-school was higher compared to during school. The only difference observed between weekend and weekdays was for sedentary time with a higher mean of overall sedentary time accumulated weekdays compared to the weekend. A significant interaction with sex was observed (p-value of interaction term: p < 0.001) with boys spending more time sedentary out of school compared to during school time, whereas there was no difference between sedentary time in-school and out-of-school for girls. There were no other statistically significant interactions with sex, SES or weight status.

**Table 2:** School-based, out-of-school and weekend physical activity (sedentary, total and vigorous-intensity physical activity) by sex# (mean ± SD; n = 1568)

	Total (n = 1568)	Boys (n = 701)	Girls (n = 867)
**Sedentary (mins)**			
School-based	236.4 (21.2)	229.1 (20.8)	242.4 (19.6)
Out-of-School	241.4 (41.7)	238.5 (42.8) ^a^	243.7 (40.5)
Weekend	420.8 (71.9) ^b, c, d^	416.6 (73.8) ^b, c, d^	424.2 (70.1) ^b, c, d^
**Total Activity (counts per minute)**			
School-based	496.4 (142.0)	536.1 (143.1)	441.5 (119.4)
Out-of-School	666.7 (257.3)^a^	697.5 (263.1) ^a^	641.4 (249.8) ^a^
Weekend	631.6 (325.0)^b, c^	670.5 (333.3) ^b, c^	599.6 (314.6) ^b, c^
**Vigorous (mins)**			
School-based	10.2 (5.4)	12.6 (5.8)	8.2 (4.0)
Out-of-School	14.9 (8.8) ^a^	16.9 (9.6) ^a^	13.1 (7.7) ^a^
Weekend	25.6 (19.1) ^b, c^	29.8 (20.8) ^b, c^	22.2 (16.8) ^b, c^

^#^The difference between boys and girls was significant for all the physical activity outcomes (P < 0.0001)

^a ^= significant difference between school-based and out-of-school physical activity (P < 0.001)

^b^= significant difference between school-based and weekend physical activity (P < 0.001)

^c ^= significant difference between out-of-school and weekend physical activity (P < 0.001)

^d ^= significant difference between weekday (combined school-based and out-of-school) and weekend physical activity (P < 0.001)

**Figure 1:**
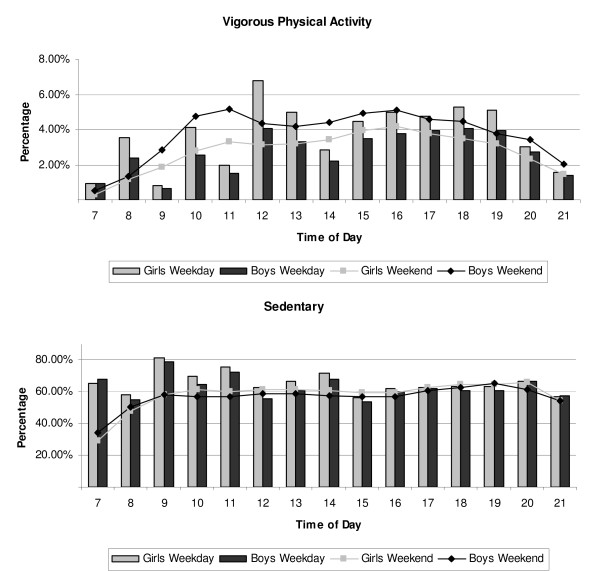
**a and b. Weekly physical activity patterns for boys and girls**. Hourly vigorous (a) and sedentary (b) physical activity patterns by weekdays (bar) and weekends (line) for boys (n = 701) and girls (n = 867).

**Figure 2:**
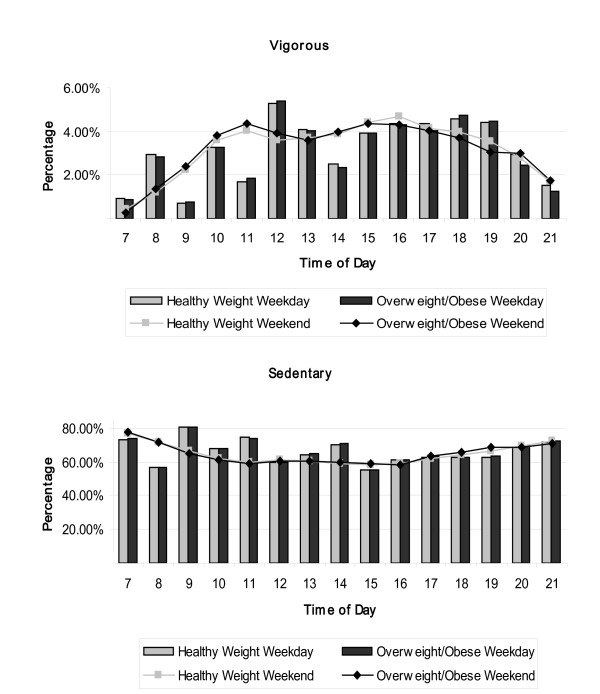
**a and b. Weekly physical activity patterns for healthy and overweight/obese children**. Hourly vigorous (a) and sedentary (b) physical activity patterns (% of measured time) for weekdays (bar) and weekends (line) for healthy weight (n = 1211) and overweight/obese (n = 357) children.

**Figure 3:**
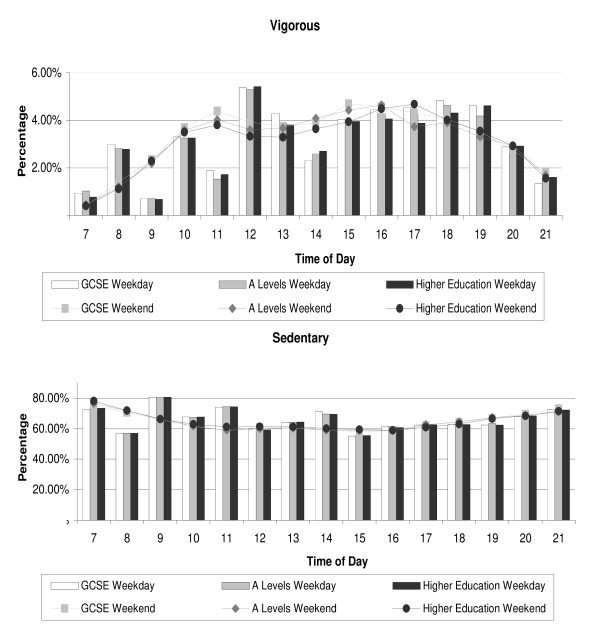
**a and b. Weekly physical activity patterns by SES**. Hourly vigorous (a) and sedentary (b) physical activity patterns (% of measured time) for weekdays (bar) and weekends (line) by SES (GCSE n = 598; A-Levels n = 644; Higher Education n = 326).

Figures 1, 2 and 3 show the hourly patterns of time spent in VPA and sedentary between 0700 h and 2100 h for weekends and weekdays by sex (Figure 1), weight status (Figure 2) and SES (Figure 3). Although girls participated in less VPA and more sedentary time, patterns during the weekday and weekends were very similar to those seen for boys. Higher volumes of VPA participation were observed before school, at break times and after school compared to lesson times. At weekends there was a steady increase in VPA across the day which started to decline after 1600 h. Similar patterns of activity were observed when the data were stratified by weight status and SES.

## Discussion

Recent literature suggests that there is a strong cross-sectional association between participation in VPA and obesity in children [9,12,25], which warrants an investigation into the potential to promote of VPA as a specific component of physical activity. One part of this may be the discouragement of sedentary activity [18]. The aim of this study was to investigate the volume and patterns of VPA and sedentary time across the segmented school week in order to identify opportunities and target groups for promoting participation in VPA. In this population of 9-10 year old children, VPA made up for about a third of the total amount of MVPA accumulated, with a maximum average of 4 minutes per hour (6% of hourly time). In contrast, approximately 35 to 50 minutes of each hour were spent in sedentary activity (60 - 80% of hourly time), both at weekdays and on weekends. This highlights that a greater emphasis needs to be placed upon finding opportunities to increase VPA, possibly at times of high sedentary activity. Public health efforts and interventions may look to mutually reduce sedentary behaviour and increase VPA and should aim to target both boys and girls regardless of weight status or SES.

When comparing out-of-school and school hours, we observed that more activity (total and VPA) was accumulated out-of-school. Boys also accumulated more sedentary time out-of-school than during school hours, whereas no difference was observed for girls. We found no difference in physical activity participation (total and VPA) between weekdays and weekends, however more sedentary time was accumulated during the week than on weekends even after adjustment for accelerometer registered wear time. From studying volumes of activity across the segmented week we conclude weekends offer an important opportunity for the promotion of VPA; when no real additional VPA compared to weekdays is being performed even though there is more flexibility for the children to engage in VPA. Others have also reported lower participation in physical activity on weekends [27-30]. The promotion of physical activity on weekends and the encouragement of spending time outdoors on weekends may be one way to increase levels of VPA [30,31]. In their longitudinal analyses, Cleland et al [31] reported that more time outdoors on weekends predicted higher MVPA on weekends among girls and boys, with the prevalence of overweight/obesity at follow-up being 27 - 41% lower among those who spent more time outdoors.

The structure of the school day and the inherent requirement to spend large amounts of time being sedentary during classes will make it more difficult to promote engaging in VPA during these times; occasions for participation in VPA are limited to breaks and physical education classes. Evidence suggests that multi-strategy school-based physical activity interventions focusing on curriculum, activity breaks and family strategies can be effective [32]. However, when examining the most effective school-based interventions, it appears that such interventions are indeed limited to structured activity through predominantly physical education (PE) classes [32] and appear to result in a net daily effect of zero; moreover they are unlikely to have an impact on body composition [33]. Further, as children progress through the educational system, participation in physical education is not always compulsory, and the contribution of school day physical activity appears to lessen as children enter secondary education [15]. Therefore identifying other avenues for the promotion of habitual lifelong participation in VPA is important.

Although there may be opportunities for promoting VPA outside of school hours [34], it appears that the children in this study are already taking this opportunity to compensate for the lower level of physical activity during the mostly sedentary school day. However, it is important to remember that these data were collected during the summer term when the weather in the UK is supportive of outdoor play and the longer hours of daylight provide the opportunity to engage in outdoor activities into the early hours of the evening. Studying these patterns during other seasons may provide a different picture.

Distinct hourly patterns were observed for sedentary time and VPA, closely following the structured school day on weekdays. More favourable physical activity patterns were observed before and after school and during break times, with relatively high amounts of sedentary time being recorded during lesson times. In support of others [e.g. [35,36]], this suggests that break times and active transportation may be important contributors to children's physical activity levels. Although boys were more physically active and girls accumulated more sedentary time, the overall patterns were similar for boys and girls, healthy weight versus overweight and obese children, or by SES, indicating that there does not appear a particular time of the week that would be more suitable to target a specific group of children. This observation is not consistent with other studies which show differences between both gender [17] and weight status [37].

The degree to which sedentary pursuits such as TV viewing and playing electronic games displaces time otherwise spent on potentially VPA remains unclear. Recent studies have shown negative associations between total physical activity and time spent TV viewing [19,38], whereas others have reported that TV viewing is unrelated to time spent participating in physical activity [39,40]. However, evidence of the co-occurrence of low levels of activity and high levels of sedentary-based activities [20], does suggest that strategies targeting both VPA and sedentary time may be necessary in the battle against obesity. Our data indicates relatively high levels of hourly sedentary behaviour across the day (≈60-80%), with low levels of VPA (0-6%). Substituting a small amount of sedentary time for vigorous time can have large population effects on levels of physical activity and children's health.

### Limitations

The limitations of accelerometry should be considered when interpreting the results of this study. For example, accelerometers do not accurately reflect activity associated with bicycling, contact sports (accelerometers are usually not worn in this situation) and swimming, or the intensity of activities such as walking up hill and upper body movement. However, objective measurement of physical activity is more accurate then other types of measures and reduces the error and bias commonly associated with self-reported measures. This measure has been extensively validated, both in the laboratory and during free-living conditions [41]. The validity of accelerometry to accurately assess sedentary time is currently under debate. We used a frequently applied cut off point (< 100 counts/min), but one must be aware that our data processing strategy of excluding data with more than 10 minutes of zero counts may have resulted in an underestimation of sedentary time. Our sample is broadly representative of the Norfolk population of school children of this age in terms of characteristics of the sampled schools compared to those of all eligible schools [23], however our sample had a lower proportion of overweight and obese children and the ethnic diversity of the Norfolk population is not representative of other parts of the UK. Although the investigation of hourly patterns of activity is a strength of this study, it may be that studying aggregated hourly patterns is still too crude. For example, morning break usually only lasts for 15 to 30 minutes and is not held at the same time at all schools. In addition, all children will have physical education lessons at different times of the day which may have affected our estimates.

## Conclusions

Based on previous evidence, it appears that specific targeted promotion of VPA in children is an important avenue in promoting health. However, levels of VPA are low and a greater understanding of physical activity patterns is needed to combat the time periods where children have low levels of activity and high amounts of sedentary time. Based on the current analyses, we conclude that promotion of VPA during the weekend may hold the greatest promise, and that interventions should target all children regardless of sex, SES or weight status. Future research should investigate this question in other seasons and include an even greater level of detail of measurement if possible. In order to be able to design effective interventions to promote VPA at times of high sedentary activity, future studies should aim to study what main behavioural activities are undertaken during VPA and sedentary time, and to investigate correlates and determinants of these behaviours.

## Competing interests

The authors declare that they have no competing interests.

## Authors' contributions

RMS was responsible for cleaning and analysing the physical activity data, conducted the data analyses and drafted the manuscript. EMFvS, UE and SJG were responsible for the overall concept, design and oversaw the collection of data for the SPEEDY study. EMFvS and UE provided critical input on the data analyses and interpretation of the results. JRL assisted in drafting the manuscript and contributed to the discussion and interpretation of the results. All authors approved the final version. None of the authors had any conflicts of interest.
